# 20-HETE Mediates Ozone-Induced, Neutrophil-Independent Airway Hyper-Responsiveness in Mice

**DOI:** 10.1371/journal.pone.0010235

**Published:** 2010-04-20

**Authors:** Philip R. Cooper, A. Clementina Mesaros, Jie Zhang, Peter Christmas, Christopher M. Stark, Karim Douaidy, Michael A. Mittelman, Roy J. Soberman, Ian A. Blair, Reynold A. Panettieri

**Affiliations:** 1 Department of Medicine and the Airways Biology Initiative, University of Pennsylvania School of Medicine, Philadelphia, Pennsylvania, United States of America; 2 Center of Excellence in Environmental Toxicology, Center for Cancer Pharmacology, University of Pennsylvania, Philadelphia, Pennsylvania, United States of America; 3 Biology Department, Radford University, Radford, Virginia, United States of America; 4 Harvard Medical School, Massachusetts General Hospital East, Charlestown, Massachusetts, United States of America; University of Alabama-Birmingham, United States of America

## Abstract

**Background:**

Ozone, a pollutant known to induce airway hyper-responsiveness (AHR), increases morbidity and mortality in patients with obstructive airway diseases and asthma. We postulate oxidized lipids mediate *in vivo* ozone-induced AHR in murine airways.

**Methodology/Principal Findings:**

Male BALB/c mice were exposed to ozone (3 or 6 ppm) or filtered air (controls) for 2 h. Precision cut lung slices (PCLS; 250 µm thickness) containing an intrapulmonary airway (∼0.01 mm^2^ lumen area) were prepared immediately after exposure or 16 h later. After 24 h, airways were contracted to carbachol (CCh). Log EC_50_ and E_max_ values were then calculated by measuring the airway lumen area with respect to baseline. In parallel studies, dexamethasone (2.5 mg/kg), or 1-aminobenzotriazol (ABT) (50 mg/kg) were given intraperitoneal injection to naïve mice 18 h prior to ozone exposure. Indomethacin (10 mg/kg) was administered 2 h prior. Cell counts, cytokine levels and liquid chromatography-mass spectrometry (LC-MS) for lipid analysis were assessed in bronchoalveolar lavage (BAL) fluid from ozone exposed and control mice. Ozone acutely induced AHR to CCh. Dexamethasone or indomethacin had little effect on the ozone-induced AHR; while, ABT, a cytochrome P450 inhibitor, markedly attenuated airway sensitivity. BAL fluid from ozone exposed animals, which did not contain an increase in neutrophils or interleukin (IL)-6 levels, increased airway sensitivity following *in vitro* incubation with a naïve PCLS. In parallel, significant increases in oxidized lipids were also identified using LC-MS with increases of 20-HETE that were decreased following ABT treatment.

**Conclusions/Significance:**

These data show that ozone acutely induces AHR to CCh independent of inflammation and is insensitive to steroid treatment or cyclooxygenase (COX) inhibition. BAL fluid from ozone exposed mice mimicked the effects of *in vivo* ozone exposure that were associated with marked increases in oxidized lipids. 20-HETE plays a pivotal role in mediating acute ozone-induced AHR.

## Introduction

Ozone, a potent oxidizing environmental pollutant, exacerbates obstructive lung diseases such as asthma and COPD, and increases hospitalization of patients [1], [2]. Ground-level ozone markedly impacts on human lung health and the US Environmental Protection Agency recently announced a stricter standard for the National Ambient Air Quality Standard for ozone at 0.075 ppm/8 h, replacing the previous limit of 0.084 ppm/8 h [3].

Controlled ozone exposure of varying durations and concentrations to humans induces airway hyper-responsiveness (AHR) commonly associated with increased airway inflammatory infiltrate [4]. Ozone-induced inflammation predominantly consists of neutrophils, that traffic, in part, to the airways due to increases in levels of cytokines and chemokines: IL-6 and IL-8 [5] among others. Studies suggest that airway sensitivity to contractile agonists is dependent on the presence of neutrophilia [6]; however, patients that develop the greatest decrements in lung function following ozone exposure do not necessarily correlate with the highest levels of neutrophilic inflammation [7], [8], [9], [10] suggesting a neutrophil independent mechanism that promotes AHR.

*In vivo* animal models of ozone-induced AHR have predominantly examined the chronic or long-term effects following ozone exposure (12–18 h) that in part focuses attention on the presence of an inflammatory infiltrate [11], [12], [13]. To date, few investigators have characterized the earliest signals after ozone exposure that mediates AHR before infiltrating inflammation occurs. Further, *in vivo* studies have exclusively examined airway functional assessments of the central airways despite evidence that the lower airways and proximal acinar regions are the most affected [14]. Though multiple protein, peptide, chemical, and lipid mediators can cause increased AHR, the identification of the specific mediator(s) of the earliest response has not been achieved.

Eicosanoids, the bioactive products of arachidonic acid (AA) are particularly intriguing candidates, as they are generated rapidly and are known to modulate AHR. These include the cyclo-oxygenase (COX)-1 and 2-dependent prostaglandins (PG) E_2_, D_2_ and F_2_, the lipoxygenase (LOX) dependent leuktrienes (LTs), or the cytochrome P450 (CYP) dependent hydroxyeicosatetraenoic acids (HETEs) or epoxyeicosatrienoic acids (EETs). Many of these eicosanoids are increased in the lung following exposure of humans to ozone [15], [16]; however, to date, whether HETEs or EETs produced via the CYP dependent pathways modulate ozone-induced AHR remains unstudied. 20-HETE, excreted primarily as a glucuronide conjugate in urine [17], is generated by the ω-hydroxylation of AA by a CYP4A enzyme found predominantly in the heart, kidney and lung [18], [19], [20]. BALB/c mice express CYP4A12 in the lung both constitutively and following IL-1β instillation [21]. CYP4A1 and 4A2 subtypes have also been localized to rat bronchial smooth muscle and epithelial cells and thus, thought to play a role in regulating airway smooth muscle tone [22]. Although the CYP4A12 is most studied source of 20-HETE, whether other CYPs contribute is unknown.

Using the precision-cut lung slice (PCLS) technique, we characterized the earliest effects of ozone exposure in mice by specifically examining the sensitivity of intra-pulmonary airways *ex vivo* at a time immediately after ozone exposure but before airway inflammatory cell infiltration. We hypothesize that ozone acutely induces AHR in a neutrophil-independent manner, through the oxidization of AA to generate 20-HETE.

## Materials and Methods

### Animals

Experiments were performed on male BALB/c mice between 8 and 12 weeks of age obtained from Charles Rivers Laboratories (Malvern, PA). Animals received water and food *ad libitum*. All mice used in this study were housed under pathogen-free conditions. The protocol was approved by the Institutional Animal Care and Use Committee of the University of Pennsylvania.

### Reagents

Carbachol (CCh), Low Melting Point Agarose (IX-A), Ham's F-12 medium (supplemented with 10% FBS, 25 mM HEPES, 2 mM glutamine, 100 U/mL penicillin, 100 µg/mL streptomycin, pH 7.6), di-isopropylethylamine (DIPEA), 2,3,4,5,6-pentafluorobenzyl bromide (PFB-Br) and solvents were purchased from Sigma-Aldrich (St. Louis, MO, USA). Heavy labeled eicosanoids and standards for quantification were purchased from Cayman Chemical Co. (Ann Arbor, MI, USA). HPLC-grade hexane, isopropanol and ethanol were obtained from Fisher Scientific Co. (Fair Lawn, NJ, USA). ACS-grade ethanol was obtained from Pharmco Products Inc. (Brookfield, CT, USA). Gases were supplied by BOC Gases (Lebanon, NJ, USA). Chiralpak AD-H column was obtained Chiral Technologies (West Chester, PA, USA). All other reagents were obtained from Sigma (St Louis, MO), unless otherwise stated.

### Ozone Exposure

Male BALB/c mice were exposed to ozone for 2 h at concentrations of either 3 ppm or 6 ppm; control mice received filtered-air for the same period of time, both groups being deprived of food and water during this time. Ozone was generated as previously described [23].

### PCLS Preparation and Airway Function Assessment

Animals were asphyxiated by carbon dioxide either immediately after ozone exposure (T = 0 h), or the following day (T = +16 h) and precision-cut lung slices (PCLS) were prepared, with slight moderations, as previously described [24], [25]. The trachea was exposed, intubated with a cannula, and the lungs were inflated with 0.65 mL 2% (w/v) low melting point agarose solution (37°C) followed by 0.1 mL bolus of air to force the agarose out of the airways and into the parenchymal tissue. After allowing the agarose to set on ice, the lobes were separated and the largest lobe was embedded externally in agarose using a tissue embedding unit (TSE systems, Chesterfield, MO). PCLS (thickness: 250 µm) were prepared using a Krumdieck tissue slicer (Alabama Research & Development Model # MD4000) with the speed set to produce slices at approximately 1 per 30 seconds. Slices were transferred in sequence to wells containing supplemented Ham's F-12 medium. Suitable airways on slices were selected on the basis of the following criteria: presence of a full smooth muscle wall (i.e., cut perpendicular to direction of airway), presence of beating cilia and internal folding of epithelium to eliminate blood vessels, and unshared muscle walls at airway branch points to eliminate possible counteracting contractile forces. Slices were then incubated at 37°C in a humidified air/CO_2_ (95:5%) incubator. Media was changed every hour for 4 h to minimize trauma and reduce airway tone as well as removing any remaining agarose in the tissue. Media was also changed first thing the following day. Up to four slices from each animal were placed in a 12 well plate in 1.0 mL buffer and were held in place using a platinum weight with nylon attachments on Day 2. Airways were located using a microscope (Nikon ECLIPSE; Model # TE2000-U; Mag.: ×100) connected to a live video feed (Evolution QEi; Model #32-0074A-130 video-recorder). A baseline image was taken (0% contraction) followed by the addition of the lowest concentration of carbachol (CCh) to begin the concentration response (10^−8^–10^−4^ M). Images were collected 4 minutes after each dose until no further contraction was evident. Previous in-house experiments have determined that this time point is sufficient to allow maximal effect at each concentration. After functional studies, the area of each airway lumen at baseline and at the end of each concentration of agonist was calculated in units of µm^2^ using Image Pro-Plus software (Media Cybernetics: Version 6.0). A log EC_50_ and E_max_ value for each airway was derived from a concentration-response curve and mean values for each animal were also derived and statistically compared using a unpaired two-tailed Student's t-test; significance was reached when P≤0.05; n-values indicate number of animals. In parallel, studies (ozone: 6 ppm; T = 0 h) were repeated following the *in vivo* administration of the steroid, dexamethasone (2.5 mg/kg), or the CYP inhibitor 1-ABT (50 mg/kg) 18 h prior to ozone exposure; or the non-selective COX-1/2 inhibitor indomethacin (10 mg/kg) 2 hr prior to ozone exposure.

### Cell Counts and KC Quantification

A bronchialalveolar lavage (BAL) was performed on carbon dioxide asphyxiated mice either immediately following ozone (6 ppm) exposure (T = 0 h), or the following day (T = +16 h). Approximately 3.0 mL was retrieved from each mouse. BAL fluid was centrifuged at 400 g (8 min, 4°C); the pellet was re-suspended in PBS and re-centrifuged. Total cell counts were obtained and slides were prepared with approximately 100,000 cells per slide. Differential cell counts were stained using Kwick™ Diff and quantified in relation to total cell counts. BAL fluid (1.0 mL) was incubated over-night with lung slices from naïve mice, followed by a concentration-response to CCh the next day (T = 0 h only), or frozen at −80°C until further needed. A DuoSet Mouse KC ELISA was carried out as per instructions of the kit (R&D Systems), samples were quantified by the comparison of unknown read-outs from a standard curve (r^2^ = 0.99). Data were expressed as mean ± SEM; n  =  number of mice.

### Oxidized Lipid Assessment

BAL fluid from animals administered dexamethasone, indomethacin or ABT and exposed to ozone (6 ppm, 2 h. T = 0 h) or filtered air was collected and oxidized lipids were measured.

#### Preparation of Eicosanoid-PFB Derivatives

The PFB derivatives were prepared by adding to the dry extract or standards in methylene chloride (100 µL), 100 µL of DIPEA in methylene chloride (1∶19, v/v) followed by 100 µL of PFB-Br in methylene chloride (1∶9, v/v) and the solution was shaken at room temperature for 30 min. The solution was evaporated to dryness under a nitrogen stream at room temperature, and re-dissolved in 100 µL of hexane/ethanol (97∶3, v/v) for mass spectrometry analysis. A 20 µL aliquot was injected each time.

#### LC

Normal-phase chiral LC was performed using a Waters Alliance 2690 HPLC system (Waters Corp., Milford, MA, USA). Gradient elution was performed in the linear mode. A Chiralpak AD-H column (250×4.6 mm i.d., 5 µm; Daicel Chemical Industries, Ltd., Tokyo, Japan) was employed with a flow rate of 1 mL/min. Solvent A was hexanes and solvent B was 2-propanol /hexanes (6/4, v/v). The linear gradient was as follows: 2% B at 0 min, 2% B at 3 min, 3.6% B at 11 min, 8% B at 15 min, 8% B at 27 min, 50% B at 30 min, 50% B at 35 min and 2% B at 37 min. The mobile phase was maintained at 30°C.

#### MS

A TSQ Quantum Ultra AM mass spectrometer (Thermo Analytical, San Jose, CA, USA) was employed for LC-MS analyses. It was equipped with an APCI) source and used in the ECAPCI negative ion mode. The operating conditions and MRM transitions were as described previously [26], [27].

#### Data Analysis

All data analysis was performed using Xcalibur software, version 2.0 SR2 (Thermo Analytical) from raw mass spectral data. Calibration curves were plotted using a linear regression of peak area ration of analytes against internal standard. Calibration curves were prepared in the range from 10 to 1000 pg/mL. Samples were stored in hexanes/2-propanol (95/5,v/v). Typical regression lines for 20-HETE (y = 0.0007x +0.0007; r^2^ = 0.983), PGE_2_ (y = 0.0014x +0.3215; r^2^ = 0.999), PGD_2_ (y = 0.0054x −0.01255; r^2^ = 0.999). Concentrations of bioactive lipids were calculated by interpolation from the calculated regression lines.

#### Eicosanoid Quantification

Assays were conducted using a modification of methods described previously [26], [27]. Briefly, a mixture of 12 heavy isotope internal standards [^2^H_8_]-5(*S*)-HETE, [^2^H_8_]-12(*S*)-HETE, [^2^H_8_]-15(*S*)-HETE, [^2^H_4_]-PGE_2_, [^2^H_4_]-PGD_2_, [^2^H_4_]-LTB_4_, [^2^H_4_]-PGF_2α_, [^2^H_4_]-11β-PGF_2_, and [^2^H_4_]-8-*iso*-PGF_2α_ (1 ng each) and [^2^H_6_]-5-oxoETE, [^2^H_6_]-20-HETE (10 ng) was added to a BAL samples (0.3 mL or 0.2 mL) followed by 1 mL of PBS 1M, pH 6.8. After standing for 10 min at room temperature to allow for equilibration, the samples were acidified to pH 4 with 5.5% formic acid, and then extracted with 75% *tert*-butylmethyl ether(TBME)/hexanes (5 mL). The organic layer was then evaporated to dryness under nitrogen, and oxidized lipids in the residue are converted to PFB derivatives. Levels of detection are less than 1.0 pg.

### CYP Enzyme Quantification by qRT-PCR

RNA was extracted from whole murine lungs following ozone exposure (6 ppm; 2 h; T = 0 h). Methods were followed as previously mentioned [28]. Briefly, reverse transcription was completed using the Applied Biosystems High-Capacity cDNA Reverse Transcription Kit (4375222) and protocol. cDNA sample was then analyzed for target gene expression of the listed CYP4a gene primer sets, with an Applied Biosystems StepOnePlus quantitative real-time PCR machine using TaqMan qPCR. Beta-Actin, mouse (4352933) was used as an endogenous control. Standard (∼2 h) reaction protocol was followed. ΔC_T_ and ΔΔC_T_ were calculated as per the manufacturer and graphed. Each sample was run in triplicate and the mean value determined. The mean (± SEM) values for the delta CTs for all experiments are shown on the graph. The following TaqMan primer sets were purchased from Applied Biosystems:

Beta-actin, CYP4a12a: Mm00514494_m1; Cyp4a12b - Mm00655431_g; H 4a14 -Mm00484132_m1; Cyp4a10 - Mm01188913_g1; Cyp4x1 - Mm01181487_m1; Cyp4a29 - Mm01188902_g1 Cyp4b1 - Mm01193710_m1.

## Results

### Ozone increases murine airway sensitivity to carbachol

**Figure 1:**
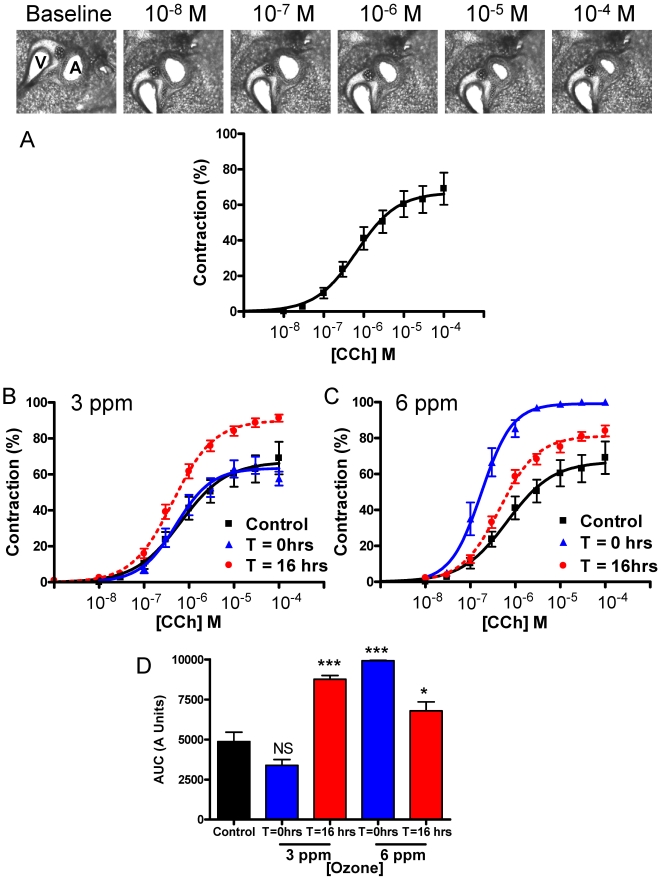
Ozone increases murine airway sensitivity to carbachol. (A) A representive contraction of a murine airway to carbachol (CCh) where A is the airway and V is the blood vessel; and a mean concentration-response curve calculated by changes in the airway lumen area with respect to the baseline image. Slices (n = 13) were prepared as described in the methods. A CCh EC_50_ value of 0.66 µM and E_max_ values of 67.1±5.0% were obtained. Airways from mice exposed to ozone at concentrations of (B) 3 ppm or (C) 6 ppm for 2 h, were also contracted to CCh. Mice were sacrificed either immediately (T = 0 h), or the next day (T = 16 h). (D) Area under the curve (AUC) units were used to statistically analyze data. Mean ± SEM shown. NS  =  Non-significant; * P≤0.05; *** P≤0.001 vs filtered air control. At least 6 animals were used in each group, and never more than 8.

To determine whether agonists induce small airway narrowing in murine PCLS, slices were incubated with cumulative doses of carbachol and luminal narrowing was then determined. Carbachol abrogated airway luminal diameter with a log EC_50_ value of −0.18±0.15 µM (EC_50_ value: 0.65 µM). Additionally, E_max_ (67.0±5.0%) of this effect was observed at a concentration of 100 µM (Figure 1A). Intra-pulmonary airways in slices from mice exposed to 3 ppm ozone exhibited an increase in airway sensitivity to carbachol when lungs were excised 16 h after the exposure (Figure 1B). The log EC_50_ or E_max_ values of murine airways from lungs excised immediately after a 3 ppm ozone exposure (T = 0 h) did not change from mice exposed to filtered air. Lungs removed 16 h after the 3 ppm ozone exposure yielded a significant increase in E_max_ and decrease in log EC_50_ values: 90.0±1.3%; P = 0.02 and −0.31±0.12 µM, P = 0.04 respectively (Figures 1B). Airways from lungs excised immediately following a 6 ppm concentration of ozone demonstrated a dramatically increase in airway responsiveness to carbachol; as shown by a significant increase in the E_max_ values from 71.8±11.1% to 99.1±0.36% (P = 0.04) and a decrease in log EC_50_ values from −0.20±0.16 µM to −0.81±0.13 µM (P = 0.01). An increase in airway sensitivity was also seen, but to a lesser extent, when airways were removed 16 h after the 6 ppm ozone exposure. Although no significant increase in E_max_ was seen despite a 19.5% change, a significant decrease in carbachol log EC_50_ values was evident: 0.08±0.13 µM vs. −0.32±0.05 µM (P = 0.01) (Figure 1C). Airway sizes were not significantly different at baseline suggesting that no airways were partially contracted before the CCh-induced contractions were carried out.

### Increases in BAL cell counts and keratinocyte-derived cytokine (KC) levels were only observed 16 h after ozone exposure

**Figure 2:**
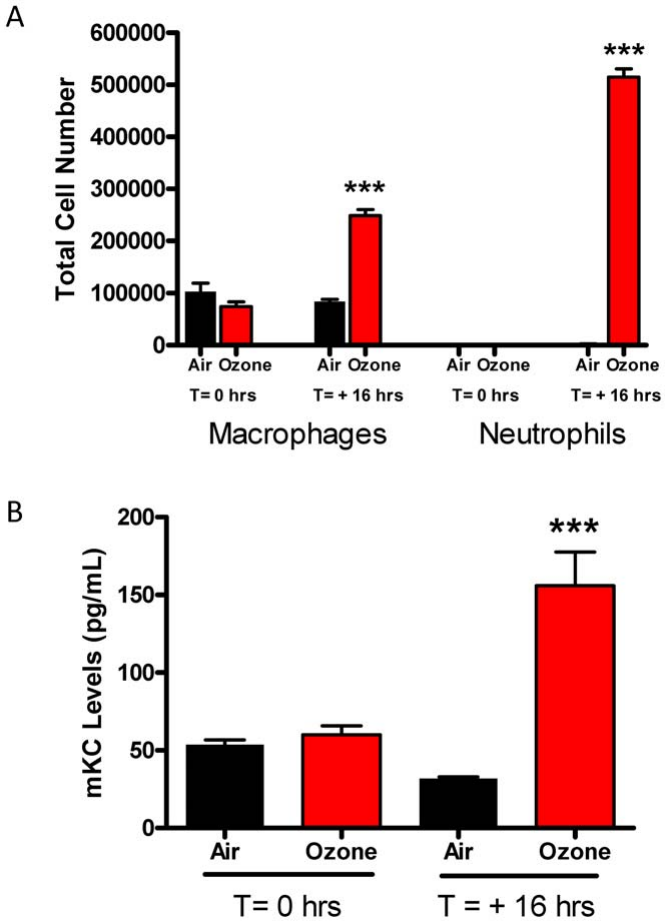
BAL fluid cell counts and KC levels following ozone exposure. Broncho-alveolar lavage (BAL) fluid was collected from mice following exposure to ozone (6 ppm; 2 h) either immediately following exposure (T = 0 h) or the following day (T = +16 h). (A) Macrophage and neutrophil cell counts; (B) mouse (m)KC levels. Data expressed as mean ± SEM shown; *** P≤0.001 vs. filtered air control.

To characterize biomarkers of ozone-induced inflammation, BAL cell profiles and chemokine levels were measured in the mice exposed to ozone or filtered air. Our previous studies [23] show that 3 ppm of ozone exposure in mice increased BAL cell counts compared to filtered air exposed mice when taken 12 h after exposure. BAL cell counts and KC levels were obtained from mice exposed to 6 ppm ozone immediately and 16 h after exposure. A 10 fold increase in total cell counts was seen in mice at 16 h after a 6 ppm ozone exposure, but no increase was seen when sacrificed immediately (data not shown). Differential cell counts identified a significant increase in both alveolar macrophages and neutrophils from BAL fluid retrieved 16 h after, but not immediately following exposure (Figure 2A). Levels of KC, the murine homolog of IL-8, paralleled the cell counts and were only elevated 16 h following 6 ppm of ozone exposure (P = 0.0005). (Figure 2B). Collectively, these data suggest that acutely after ozone exposure there exists no perceivable airway inflammation but profound AHR.

### BAL fluid from ozone-exposed mice induces AHR in slices from naïve mice

**Figure 3:**
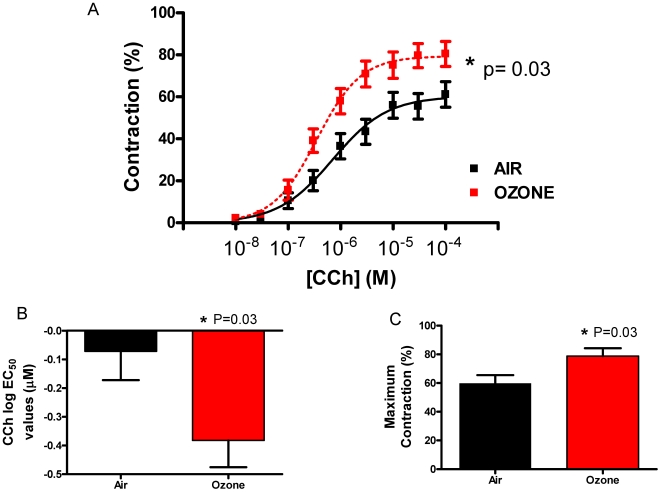
BAL fluid from ozone-exposed mice induces AHR in slices from naïve mice. Lung slices from naïve mice were incubated with BAL fluid from mice exposed to ozone (6 ppm; 2 h; T = 0 h), or filtered air. (A) Airways were contracted to carbachol (CCh) to create a concentration-response curve. BAL fluid from ozone-exposed mice (B) decreased log EC_50_ values; and, (C) increased maximum contraction compared to slices incubated with BAL fluid from air-exposed mice. Data expressed as mean ± SEM shown; * P≤0.05 vs. filtered air control. At least 14 airways were used in each group, and never more than 19.

To determine whether soluble mediators in BAL fluid induce AHR, 1.0 mL BAL fluid from mice exposed to ozone (6 ppm; 2 h, T = 0 h) was incubated overnight with lung slices from naïve mice. BAL fluid from mice exposed to 6 ppm ozone immediately following exposure manifested little inflammation but exhibited AHR; we postulated that a mediator in BAL may induce AHR. Lung slices were also incubated with BAL from filtered air-exposed mice. Following incubation with BAL from ozone-exposed mice, murine airways in naïve slices demonstrated an increase in airway sensitivity, as shown by a decrease in log EC_50_ values: −0.07±0.10 µM vs. −0.38±0.09 µM (P = 0.03); and an increase in E_max_ values: 59.9±5.7% vs. 78.8±5.5% (P = 0.03) (Figure 3).

### Eicosanoid production in murine BAL following ozone exposure

**Figure 4:**
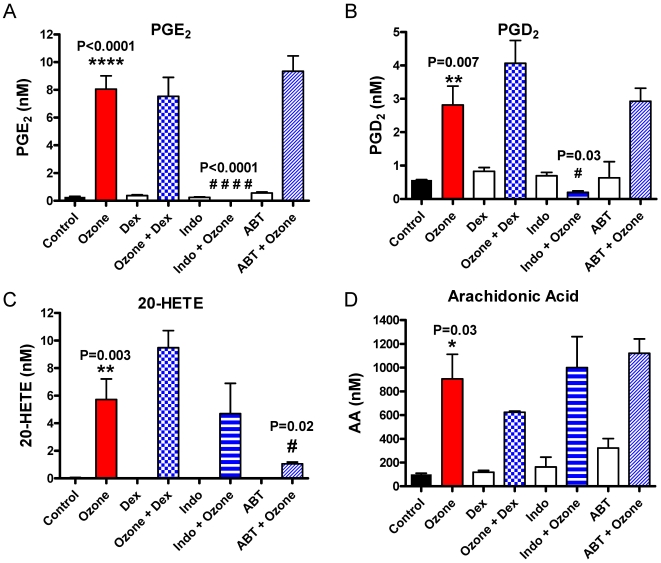
Ozone increases oxidized lipids levels in the BAL fluid. Mice were administered either dexamethasone (2.5 mg/kg) or ABT (50 mg/kg) 18 h prior or indomethacin (10 mg/kg) 2 hr prior to ozone (6 ppm, 2 h; T = 0 h) exposure, with control mice exposed to filtered air. BAL fluid was retrieved and analyzed for (A) PGE_2_, (B) PGD_2_, (C) 20-HETE and (D) AA as previously described. Data is expressed as mean ± SEM shown. **** P<0.001 vs. control; ** P≤0.01 vs. control; # P≤0.05 vs. ozone. At least 3 animals were used in each group, and never more than 7.

**Figure 5:**
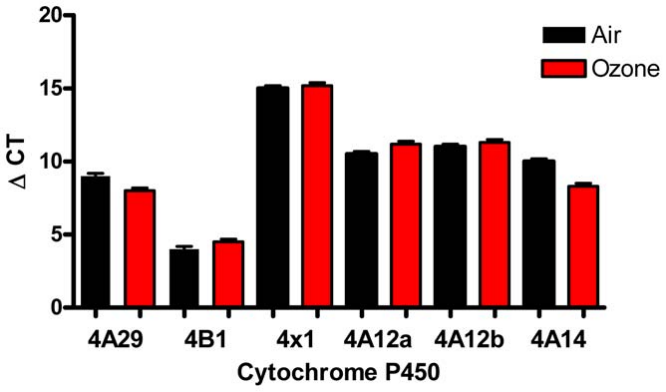
Ozone did not increase CYP enzymes measured. mRNA was extracted from whole lungs of mice exposed to ozone (6 ppm; 2 h; T = 0 h) and CYP isotypes were quantified using Taq Man qPCR, as described in the methods. Data was run in triplicate and expressed as mean ± SEM (n = 5 in each group).

Initially, the hypothesis that a cytokine, chemokine or growth factor known to increase AHR was released into the BAL fluid in response to ozone was tested. Surprisingly, levels of IL-6, IL-13 and transforming growth factor (TGF)-β were un-altered in the BAL fluid from control and ozone treated lungs. Eicosanoids thought to be potential mediators for AHR were then measured. PFB derivatives of eicosanoids derived from AA were analyzed under electron capture atmospheric chemcical ionization (ECAPCI/MS) conditions. The LC-ECAPCI/multiple reaction monitoring *MRM)/MS profile for AA metabolites from control BAL samples (animals exposed to filtered air) revealed the presence of 5(*R*)-HETE (1.47 nM), 5(*S*)-HETE (1.00 nM), 12(*S*)-HETE (0.47 nM), 15(*R*)-HETE (0.05 nM) and 15(*S*)-HETE (1.32 nM), 11(*R*)-HETE (0.84 nM) and 11(*S*)-HETE (1.01 nM). There was a trace amount of 12(*R*)-HETE but 20-HETE, LTB_4_, 11β-PGF_2_ TBX_2_ (thromboxane) and 8-*iso*-PGF_2α_ were undetectable in the control BAL samples. PGE_2_, PGD_2_, and PGF_2α_ were present at 0.28 nM, 0.57 nM, and 0.32 nM, respectively in the same control samples. When the animals were exposed to 6 ppm of ozone the levels of most of the eicosanoids increased (Table 1). LTB_4_, TBX_2_ and 11β-PGF_2_, however, were still below the limit of detection. The levels of 20-HETE increased dramatically from non-detectable levels in the air treated animals to a concentration of 1.83 ng/mL (5.7 nM) in the animals exposed to ozone (P = 0.003) (Figure 4 & Table 1). Ozone-treatment increased all of the other HETEs in the BAL except for 12(*R*)-HETE (Table 1), PGE_2_ and PGF_2α_ concentrations increased substantially in the BAL of ozone-treated animals from 0.08 ng/mL (0.28 nM) and 0.11 ng/mL (0.32 nM), respectively to 2.84 ng/mL (8.06 nM) and 4.00 ng/mL (11.3 nM), respectively (Figure 4, Table 1). In contrast, there was a modest increase in PGD_2_ concentrations from 0.20 ng/mL (0.57 nM) to 1.00 ng/mL (2.81 nM). Dexamethasone, indomethacin, and ABT had little effect on eicosanoid content of control BAL (Table 1). When animals, however, were treated with indomethacin 2 h prior to ozone exposure, the levels of PGE_2_, and PGF_2α_ decreased to undetectable levels and PGD_2_ decreased to, 0.19 nM. There was no affect on 20-HETE (4.7 nM). In contrast, pre-treatment of the animals with ABT prior to ozone exposure induced an almost 6-fold reduction in the levels of 20-HETE from 5.72 nM to 1.05 nM, but had no significant effect on the other eicosanoids (Table 1). Total AA was also measured and was increased almost 10 fold. None of the pharmacological agents had an effect on total AA (Figure 4D). To analyze the effect of ozone on CYP mRNA expression, RNA was extracted from whole murine lungs following ozone exposure (6 ppm; 2 h; T = 0 hrs) and analyzed by a two step procedure. RNA was then analyzed by conventional RT-PCR to determine which members of the CYP4A family were represented. In parallel RNA was also analyzed by TaqMan qPCR. As shown in figure 5, both CYP4a12 isoforms A and B were identified in relatively equal amounts, and RNA levels were not significantly affected by ozone exposure.

**Table 1:** Level of eicosanoids in murine BAL fluid following ozone exposure.

Eicosanoid	Air	Ozone	Dex	Dex + Ozone	Indo	Indo + Ozone	ABT	ABT + Ozone
**PGE_2_**	0.28±0.06	8.06±0.96	0.37±0.06	7.54±1.37	0.24±0.04	ND	0.56±0.10	9.36±2.22
**PGD_2_**	0.57±0.01	2.81±0.56	0.83±0.16	4.07±0.69	0.70±0.09	0.19±0.09	0.63±0.06	1.77±0.73
**PGF_2α_**	0.32±0.30	11.29±0.35	0.23±0.02	13.4±0.30	0.28±0.10	ND	0.21±0.07	12.12±2.60
**20-HETE**	0.04±0.02	5.72±1.48	ND	9.48±1.24	ND	4.70±2.49	ND	1.05±0.25
**5(** ***R*** **)-HETE**	1.47±0.23	5.15±1.13	1.63±0.54	6.09±3.50	1.43±0.91	0.12±0.03	1.42±0.51	5.04±1.69
**5(** ***S*** **)-HETE**	1.00±0.18	3.34±0.94	1.24±0.29	4.98±2.40	1.02±0.61	0.26±0.02	0.90±0.55	4.03±2.09
**12(** ***R*** **)-HETE**	≤0.001	0.004	≤0.001	0.008	≤0.001	ND	≤0.001	0.005
**12(** ***S*** **)-HETE**	0.47±0.15	1.37±0.37	0.76±0.12	2.64±1.24	0.47±0.23	4.07±1.87	0.51±0.17	1.89±1.22
**15(** ***R*** **)-HETE**	0.06±0.02	3.52±1.74	1.02±0.36	2.30±0.23	0.51±0.37	0.30±0.17	0.09±0.01	3.50±2.31
**15(** ***S*** **)-HETE**	1.32±0.60	4.25±2.01	0.86±0.13	5.59±3.37	0.76±0.09	0.40±0.12	0.21±0.17	4.64±3.2
**11(** ***R*** **)-HETE**	0.84±0.27	3.52±1.65	0.28±0.07	3.99±0.52	≤0.001	0.20±0.16	≤0.001	3.95±2.23
**11(** ***S*** **)-HETE**	1.01±0.35	1.52±0.45	1.67±0.20	2.46±1.04	0.80±0.15	0.05±0.07	0.53±0.35	2.05±0.59
**TBX_2_**	ND	ND	ND	ND	ND	ND	ND	ND

Mice were administered either dexamethasone (2.5 mg/kg) or ABT (50 mg/kg) 18 h prior or indomethacin (10 mg/kg) 2 hr prior to ozone (6 ppm, 2 h; T = 0 h) exposure, with control mice exposed to air. BAL fluid was retrieved and analyzed for eicosanoids using mass spectrometry. Data (n = 4) is expressed as mean ± SEM (nM). ND  =  Not detected.

### CYP inhibition, but not dexamethasone nor indomethacin attenuates ozone-induced AHR

**Figure 6:**
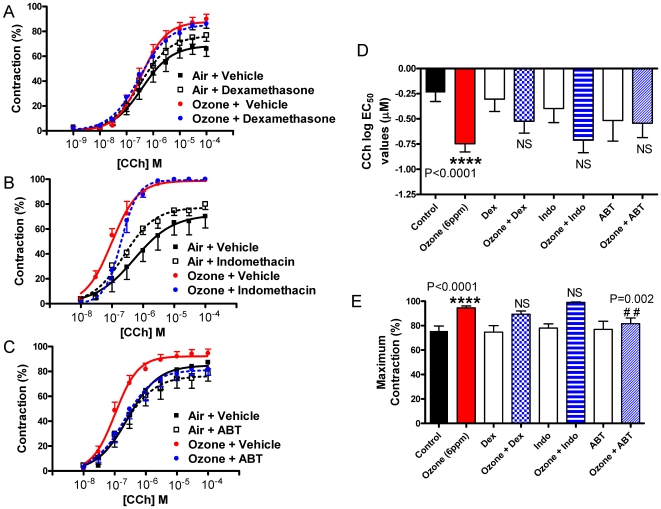
CYP inhibition abrogates ozone-induced increases in airway sensitivity. Mice were administered either (A) dexamethasone (2.5 mg/kg; 18 h prior); (B) indomethacin (10 mg/kg; 2 hr prior); or (C) ABT (50 mg/kg; 18 hr prior) to ozone (6 ppm, 2 h; T = 0 h) exposure, with control mice exposed to filtered air. Lung slices were prepared, and airways were contracted to carbachol (CCh) to create concentration response curves, from which (D) log EC_50_ values and (E) maximum contractions were calculated. Data is expressed as Mean ± SEM shown. NS  =  Non-significant; **** P<0.001 vs. control; # # P≤0.01 vs. ozone. At least 6 animals were used in each group, and never more than 10.

To determine whether CYP activity was required to induce eicosanoid-mediated AHR, animals were administered dexamethasone, ABT (18 h) or indomethacin (2 h) prior to ozone exposure (6 ppm; 2 h; T = 0 h). Ozone exposure caused a leftward shift in the concentration-response curve as shown in Figure 6A–C. The pre-administration of dexamethasone had little effect on the ozone-induced increase in airway sensitivity; nor did the pre-administration of indomethacin. Our previous studies have shown that this concentration and time course of dexamethasone inhibited allergen-induced airway inflammation in mice (data unpublished), and the current data also showed significant PG inhibition following indomethacin administration (Figure 4A). The CYP inhibitor, ABT, however completely abrogated the effects of ozone on CCh-induced airway luminal airway narrowing. (E_max_ values (ozone: 94.6±1.5% vs. ozone + ABT: 81.7±4.7% (P = 0.002)). In none of the 3 experiments did the drug in question have an effect on air-exposed animals.

### 20-HETE increases murine airway sensitivity to carbachol

**Figure 7:**
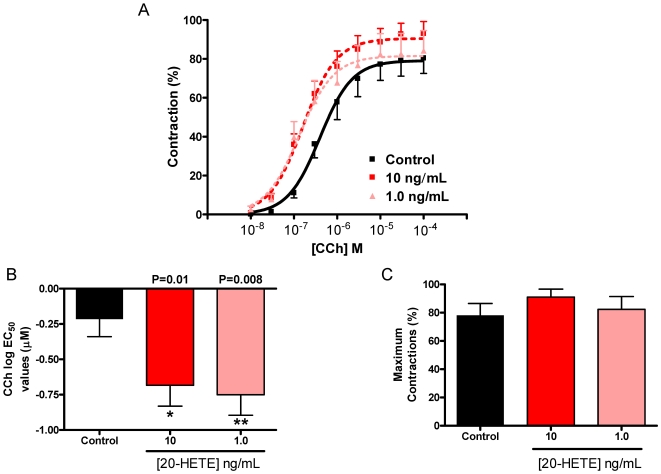
20-HETE increases murine airway sensitivity to carbachol. Lung slices from naïve mice were incubated over-night with 20-HETE (10 and 1 ng/mL). (A) Airways were contracted to carbachol (CCh) to create concentration-response curves. Incubation with 20-HETE (B) decreased log EC_50_ values; but, (C) had no effect on maximum contraction compared to control slices. Data expressed as mean ± SEM shown; * P≤0.05 and ** P≤0.01 vs control (exact P values shown). At least 8 airways were used in each group, and never more than 12.

Having demonstrated that the inhibition of the production of 20-HETE augmented the ozone-induced AHR, lung slices containing an airway from naïve mice were incubated over-night with 20-HETE (0, 1.0 and 10 ng/mL/0, 3.1, 31.0 nM) to determine whether 20-HETE mediates AHR. Airways were contracted to carbachol. As shown in figure 7, 20-HETE increased the maximum bronchoconstrction and increased airway sensitivity by 16% in the E_max_ and significantly decreased in log EC_50_ values compared to airways incubated in the absence of 20-HETE. A lower concentration of 20-HETE (1 ng/mL) significantly increased airway sensitivity without increasing the maximum contraction. 20-HETE (20 ng/mL/62 nM) alone had little effect on basal bronchomotor tone in naïve slices.

## Discussion

Although ozone induces exacerbations of asthma and COPD, and has also been implicated in accelerating cardiovascular disease, the precise cellular and molecular mechanisms are poorly described. Compelling evidence suggests that ozone induces airway inflammation manifested by increases in neutrophil number and levels of tumor necrosis factor-α and IL-1β. To date, the role of inflammation in mediating ozone-induced AHR remains controversial. Further, studies suggest uncoupling of airway inflammation and AHR after ozone exposure in humans [7], [8], [9], [10].

The PCLS technique serves as a suitable model to measure ozone-induced AHR *ex vivo* as demonstrated by an increase in airway sensitivity to a muscarinic agonist following ozone exposure (3 ppm; 2 h; T = +16 h: Figure 1B), compared to air-matched controls. Increased levels of neutrophils were also evident. This was previously shown in a model of pulmonary resistance and was associated with an increase in trafficking cells in the BAL fluid, specifically neutrophils [23]. Following exposures of the same concentration of ozone, but at a time where no infiltration cells were present (3 ppm; 2 h; T = 0 h: Figure 1B); no change in airway sensitivity compared to air-controls was observed. After mice were exposed to increased concentrations of ozone (6 ppm), at a time of increased neutrophils in the BAL fluid an increase in airway sensitive to carbachol was observed (6 ppm; 2 h; T = +16 h: Figure 1C). Interestingly, when the time between ozone exposure and animal sacrifice was shortened from 16 to 0 h, AHR was still evident despite no increase in total cell number, neutrophils and KC levels, in the BAL fluid. Despite studies specifically examining neutrophil-dependent AHR [12], [13], only a few *in vivo* rodent [29], [30], and human studies [8], [10] have also noted an increase in airway sensitivity lacking a correlation with early airway inflammation. One study exposed neutrophil-depleted rats to ozone (1.0 ppm) for 8 h and concluded that neutrophils did not play a significant role in the acute ozone-induced early epithelial damage seen in non-neutrophil-depleted rats [31]. One must therefore ask if an ozone-induced increase in IL-8 that attracts neutrophils into the lung is responsible for the latent AHR; what mechanism is causing the early phase AHR seen in our study? To determine whether the inhalation of ozone induced the release of a non-immunological mediator, or was directly modulating airway smooth muscle function rendering it more contractile; BAL fluid from mice exposed to ozone (6 ppm; 2 h; T = 0 h) or filtered air was incubated overnight on lung slices from naive mice. Airways on naïve lung slices incubated with BAL fluid from ozone-exposed mice were more responsive and more sensitive to carbachol, as defined by an increase in the maximum contraction and a decrease in the log EC_50_ values respectively, than those incubated with BAL fluid from air-exposed mice. These data suggest that ozone promotes AHR via secretion of a soluble mediator from the airway into the BAL fluid.

Eicosinoids are increased in the lungs following ozone exposure [15], [16], [32]. Alfaro *et al.* reported that subjects with more sensitive airways had a greater amount of PGE_2_, 8-*iso*-PGF_2α_ and LTD_4_ in the exhaled breath condensate than subjects with less sensitive airways following an acute exposure to ozone [16]. In the present study, BAL fluid from mice exposed to ozone (6 ppm, 2 h, T = 0 h) was analyzed for quantities of oxidized lipids. Our targeted lipidomics approach for the analysis of BAL fluid revealed that 20-HETE levels were significantly increased upon exposure of mice to ozone. These data suggest that either CYP4A isoforms, responsible for ω-hydroxylation of AA, were up-regulated by ozone treatment [33], or that increases in AA substrate was generated leading to increases in 20-HETE, or that a combination of these mechanisms were in play. When the cytochrome P450 inhibitor ABT was administered 18 h prior to exposure to ozone, the levels of 20-HETE in BAL were reduced by 82%. This is similar to the 84% reduction of renal cortical 20-HETE formation observed in Sprague-Dawley rats treated with similar levels of ABT [34]. The decreased concentrations of 20-HETE in BAL fluid coincided with the complete reversal of the ozone-induced increase in airway sensitivity, suggesting that 20-HETE production played an important role in the AHR when no infiltrating inflammatory cells or mediators were present. The lack of attenuation of the other HETEs suggests the specificity of ABT to 20-HETE in the murine model. Studies have suggested that 1-ABT is a non-selective inhibitor of CYP enzymes [35]; however, the same concentration as was used in the present study demonstrated a fairly selective inhibition of the CYP4A/CYP4F catalyzed formation of 19-and 20-HETE in Sprague Dawley kidneys with no inhibition of epoxygenases [34]. In the present study, no other HETEs were inhibited following 1-ABT administration suggesting it to be selective to the oxidized lipids that were measured. Future studies may warrant the use of the more selective 20-HETE synthesizing enzyme inhibitor HET0016 [35]. PGE_2_ and PGF_2α_ were two of the other the most abundant eicosanoids detected in the BAL after ozone treatment (Figure 4) and are known to induce AHR; however, they were still present despite the ABT-induced decrease in airway sensitivity. The non-selective COX-inhibitor, indomethacin reduced the levels of PGE_2_, PGF_2α_, and PGD_2_ levels by 100%, 100%, and 93%, respectively (Table 1) but had no effect on the ozone-induced increase in airway sensitivity, suggesting that PGs do not play a significant role in this model of AHR. Indeed, other studies have shown no evidence of indomethacin inhibiting early phase ozone-induced airway hyper-responsiveness, and have suggested other arachidonic acid metabolites to be responsible for the increase in airway sensitivity [36], [37]. However, Nakando and colleagues demonstrated an increase in neutrophils immediately after ozone exposure, suggesting that neutrophil-dependent AHR was evident, that may be independent of AHR induced by increases in oxidized lipid products in their study. Hazucha and colleagues also demonstrated a lack of an effect with COX inhibition on an ozone-induced increase of airway resistance in human subjects, when PGE_2_ and thromboxane were reduced [38]; however, like Nakando, but unlike the present study, neutrophils were also present. In addition, ozone exposure of mice deficient in PGE-synthase, which is needed for the generation of PGE_2_, had little effect on AHR (unpublished data). Dexamethasone pre-treatment had no effect on the oxidized lipids in the BAL, or on the ozone-induced AHR. This could, in part, be due to steroids having little or no effect on models of oxidative stress [39], [40]. LOX inhibitors were not used as levels of LTs were not shown to be increased following the exposure of ozone, thus not thought to play a role in the AHR seen in the present study. Thromboxane levels were not detected following any condition.

Collectively, our data suggests that 20-HETE plays a significant role in the neutrophil-independent AHR because the inhibition of 20-HETE production by ABT attenuated ozone-induced increases in airway sensitivity, when other HETEs or PGs were not affected. Previous studies have suggested that 20-HETE modulates airway tone [22], [41], [42]; however, our study failed to show evidence of the direct effect on airway contraction in murine airways. Incubation of 20-HETE with murine lung slices, however, increased its contractile sensitivity to carbachol as compared with diluent treated slices. Concentrations of 20-HETE used were comparable to those measured in the BAL fluid following ozone exposure. Concentrations 100 fold lower than those measured in the BAL fluid were also shown to increase sensitivity of airway contraction. The mechanism of 20-HETE-induced AHR is unknown at this time and warrants further investigation. Although the slices are exposed to ozone for 2 hours, airway functional assessment is not carried out until the next day. This is due to a limitation of the preparation in that although lung slices are prepared immediately, the removal of agarose and the allowance of time for the airway to revert back to basal tone following slicing is required. Within this time, the effects of the ozone exposure are still at play at the intra-cellular level. Therefore, to mimic this as close as possible, 20-HETE and BAL fluid was incubated over-night to time-match the *ex vivo* study.

To date, only CYP4A12a and 4A12b have been shown to generate 20-HETE from AA [43]. In humans, CYP4F2 and CYP4F3B play a major role in generating 20-HETE. In mice, however, members of the CYP4F family modify the ω-, and ω-1 positions of AA to generate 18-HETE and 19-HETE. Therefore, though we determined the relative amounts of murine CYP4A family members and their relative change in response to ozone, we focused predominantly on the relative expression of the isoforms CYP4A12a and CYP4A12b in response to ozone. As shown in figure 5, CYP4A12a and CYP4A12b expression, as determined by qRT-PCR, were similar. Ozone had little effect on the relative expression of either gene product. Therefore the induction of 20-HETE must be regulated at a step proximal to CYP4A expression and is likely due to increases in substrate delivery by increased generation of AA via cPLA_2_ activity or at the re-acylation of AA into membrane phospholipids as previously described [44]. Levels of AA were increased in murine BAL fluid following ozone exposure (Figure 6D), therefore providing increased levels of substrate for the CYP enzymes to convert to 20-HETE. Surprisingly, not all oxidized lipid mediators downstream from AA were increased such as the LTs, suggesting that CYP enzyme activity may be another mechanism modulating AHR following ozone exposure. Macrophages present in the lung tissue may also be a source of 20-HETE production.

It is worth emphasizing that the current study is taking a snap-shot immediately after ozone exposure, eliminating infiltrating inflammation by creating the slices, and hypothesizing that a CYP4A dependent pathway is (directly or indirectly) responsible for the AHR shown. A limitation of the current study is that we cannot rule out the exact mechanism as a time-course of the supernatants post-ozone post-slicing was not carried out; however, an advantage is that we can eliminate the role of infiltrating cells. This will lead us to our follow-up studies.

Although we recognize the concentration of ozone needed to achieve the phenomenon of neutrophil-independent AHR is greater than that defined by the EPA to be hazardous to humans [3], the fractional deposition of ozone in mice is only 25% compared to 100% for humans. The EPA also recognizes an exposure time of 8 h which is much longer than the 2 h duration in the present study. Our data suggests that 20-HETE generated by ozone may represent one of the earliest and most robust signals promoting ozone-induced AHR.

In summary, eicosinoids released into the airway increased following ozone that was not attenuated by dexamethasone or indomethacin. Only inhibition of 20-HETE production attenuated both the ozone-induced elevated levels 20-HETE and AHR. We postulate that the increased 20-HETE is derived from an increase in AA, as well as the possibility of increased CYP activity, but not by an increase expression of CYP enzymes. Modulation of the production of 20-HETE may serve as a new therapeutic target to prevent ozone-induced exacerbations of asthma and COPD or serve as an important biomarker of oxidative stress in the airways.

## References

[1] Galan I, Tobias A, Banegas JR, Aranguez E (2003). Short-term effects of air pollution on daily asthma emergency room admissions.. Eur Respir J.

[2] Medina-Ramon M, Zanobetti A, Schwartz J (2006). The effect of ozone and PM10 on hospital admissions for pneumonia and chronic obstructive pulmonary disease: a national multicity study.. Am J Epidemiol.

[3] Stokstad E (2008). Air-quality standards. EPA adjusts a smog standard to White House preference.. Science.

[4] Ratto J, Wong H, Liu J, Fahy J, Boushey H (2006). Effects of multiday exposure to ozone on airway inflammation as determined using sputum induction.. Environ Health Perspect.

[5] Jorres RA, Holz O, Zachgo W, Timm P, Koschyk S (2000). The effect of repeated ozone exposures on inflammatory markers in bronchoalveolar lavage fluid and mucosal biopsies.. Am J Respir Crit Care Med.

[6] Seltzer J, Bigby BG, Stulbarg M, Holtzman MJ, Nadel JA (1986). O3-induced change in bronchial reactivity to methacholine and airway inflammation in humans.. J Appl Physiol.

[7] Arjomandi M, Witten A, Abbritti E, Reintjes K, Schmidlin I (2005). Repeated exposure to ozone increases alveolar macrophage recruitment into asthmatic airways.. Am J Respir Crit Care Med.

[8] Blomberg A, Mudway IS, Nordenhall C, Hedenstrom H, Kelly FJ (1999). Ozone-induced lung function decrements do not correlate with early airway inflammatory or antioxidant responses.. Eur Respir J.

[9] Schelegle ES, Siefkin AD, McDonald RJ (1991). Time course of ozone-induced neutrophilia in normal humans.. Am Rev Respir Dis.

[10] Balmes JR, Chen LL, Scannell C, Tager I, Christian D (1996). Ozone-induced decrements in FEV1 and FVC do not correlate with measures of inflammation.. Am J Respir Crit Care Med.

[11] Williams AS, Issa R, Leung SY, Nath P, Ferguson GD (2007). Attenuation of ozone-induced airway inflammation and hyper-responsiveness by c-Jun NH2 terminal kinase inhibitor SP600125.. J Pharmacol Exp Ther.

[12] Williams AS, Leung SY, Nath P, Khorasani NM, Bhavsar P (2007). Role of TLR2, TLR4, and MyD88 in murine ozone-induced airway hyperresponsiveness and neutrophilia.. J Appl Physiol.

[13] Jang AS, Choi IS, Lee JH, Park CS, Park CS (2006). Prolonged ozone exposure in an allergic airway disease model: adaptation of airway responsiveness and airway remodeling.. Respir Res.

[14] Bascom R, Bromberg P, Costa D (1996). Health effects of outdoor air pollution. Committee of the Environmental and Occupational Health Assembly of the American Thoracic Society.. Am J Respir Crit Care Med.

[15] Chen C, Arjomandi M, Balmes J, Tager I, Holland N (2007). Effects of chronic and acute ozone exposure on lipid peroxidation and antioxidant capacity in healthy young adults.. Environ Health Perspect.

[16] Alfaro MF, Walby WF, Adams WC, Schelegle ES (2007). Breath condensate levels of 8-isoprostane and leukotriene B4 after ozone inhalation are greater in sensitive versus nonsensitive subjects.. Exp Lung Res.

[17] Prakash C, Zhang JY, Falck JR, Chauhan K, Blair IA (1992). 20-Hydroxyeicosatetraenoic acid is excreted as a glucuronide conjugate in human urine.. Biochem Biophys Res Commun.

[18] Wu S, Chen W, Murphy E, Gabel S, Tomer KB (1997). Molecular cloning, expression, and functional significance of a cytochrome P450 highly expressed in rat heart myocytes.. J Biol Chem.

[19] Zhu D, Effros RM, Harder DR, Roman RJ, Jacobs ER (1998). Tissue sources of cytochrome P450 4A and 20-HETE synthesis in rabbit lungs.. Am J Respir Cell Mol Biol.

[20] Imig JD, Zou AP, Stec DE, Harder DR, Falck JR (1996). Formation and actions of 20-hydroxyeicosatetraenoic acid in rat renal arterioles.. Am J Physiol.

[21] Le Bouquin R, Lugnier A, Frossard N, Pons F (2004). Expression of cytochrome P450 4A mRNA in mouse lung: effect of clofibrate and interleukin-1beta.. Fundam Clin Pharmacol.

[22] Zhu D, Zhang C, Medhora M, Jacobs ER (2002). CYP4A mRNA, protein, and product in rat lungs: novel localization in vascular endothelium.. J Appl Physiol.

[23] Kierstein S, Krytska K, Sharma S, Amrani Y, Salmon M (2008). Ozone inhalation induces exacerbation of eosinophilic airway inflammation and hyperresponsiveness in allergen-sensitized mice.. Allergy.

[24] Held HD, Martin C, Uhlig S (1999). Characterization of airway and vascular responses in murine lungs.. BrJPharmacol.

[25] Henjakovic M, Martin C, Hoymann HG, Sewald K, Ressmeyer AR (2008). Ex vivo lung function measurements in precision-cut lung slices (PCLS) from chemical allergen-sensitized mice represent a suitable alternative to in vivo studies.. Toxicol Sci.

[26] Lee SH, Blair IA (2007). Targeted chiral lipidomics analysis by liquid chromatography electron capture atmospheric pressure chemical ionization mass spectrometry (LC-ECAPCI/MS).. Methods Enzymol.

[27] Mesaros C, Lee SH, Blair IA (2009). Targeted quantitative analysis of eicosanoid lipids in biological samples using liquid chromatography-tandem mass spectrometry.. J Chromatogr B Analyt Technol Biomed Life Sci.

[28] Christmas P, Tolentino K, Primo V, Berry KZ, Murphy RC (2006). Cytochrome P-450 4F18 is the leukotriene B4 omega-1/omega-2 hydroxylase in mouse polymorphonuclear leukocytes: identification as the functional orthologue of human polymorphonuclear leukocyte CYP4F3A in the down-regulation of responses to LTB4.. J Biol Chem.

[29] Depuydt P, Joos GF, Pauwels RA (1999). Ambient ozone concentrations induce airway hyperresponsiveness in some rat strains.. Eur Respir J.

[30] Evans TW, Brokaw JJ, Chung KF, Nadel JA, McDonald DM (1988). Ozone-induced bronchial hyperresponsiveness in the rat is not accompanied by neutrophil influx or increased vascular permeability in the trachea.. Am Rev Respir Dis.

[31] Pino MV, Stovall MY, Levin JR, Devlin RB, Koren HS (1992). Acute ozone-induced lung injury in neutrophil-depleted rats.. Toxicol Appl Pharmacol.

[32] Schlesinger RB, Driscoll KE, Gunnison AF, Zelikoff JT (1990). Pulmonary arachidonic acid metabolism following acute exposures to ozone and nitrogen dioxide.. J Toxicol Environ Health.

[33] Kroetz DL, Xu F (2005). Regulation and inhibition of arachidonic acid omega-hydroxylases and 20-HETE formation.. Annu Rev Pharmacol Toxicol.

[34] Su P, Kaushal KM, Kroetz DL (1998). Inhibition of renal arachidonic acid omega-hydroxylase activity with ABT reduces blood pressure in the SHR.. Am J Physiol.

[35] Miyata N, Taniguchi K, Seki T, Ishimoto T, Sato-Watanabe M (2001). HET0016, a potent and selective inhibitor of 20-HETE synthesizing enzyme.. Br J Pharmacol.

[36] Nakano H, Aizawa H, Matsumoto K, Fukuyama S, Inoue H (2000). Cyclooxygenase-2 participates in the late phase of airway hyperresponsiveness after ozone exposure in guinea pigs.. Eur J Pharmacol.

[37] Lee HK, Murlas C (1985). Ozone-induced bronchial hyperreactivity in guinea pigs is abolished by BW 755C or FPL 55712 but not by indomethacin.. Am Rev Respir Dis.

[38] Hazucha MJ, Madden M, Pape G, Becker S, Devlin R (1996). Effects of cyclo-oxygenase inhibition on ozone-induced respiratory inflammation and lung function changes.. Eur J Appl Physiol Occup Physiol.

[39] Marwick JA, Ito K, Adcock IM, Kirkham PA (2007). Oxidative stress and steroid resistance in asthma and COPD: pharmacological manipulation of HDAC-2 as a therapeutic strategy.. Expert Opin Ther Targets.

[40] Ito K, Herbert C, Siegle JS, Vuppusetty C, Hansbro N (2008). Steroid-resistant neutrophilic inflammation in a mouse model of an acute exacerbation of asthma.. Am J Respir Cell Mol Biol.

[41] Cloutier M, Campbell S, Basora N, Proteau S, Payet MD (2003). 20-HETE inotropic effects involve the activation of a nonselective cationic current in airway smooth muscle.. Am J Physiol Lung Cell Mol Physiol.

[42] Morin C, Sirois M, Echave V, Gomes MM, Rousseau E (2007). Functional effects of 20-HETE on human bronchi: hyperpolarization and relaxation due to BKCa channel activation.. Am J Physiol Lung Cell Mol Physiol.

[43] Muller DN, Schmidt C, Barbosa-Sicard E, Wellner M, Gross V (2007). Mouse Cyp4a isoforms: enzymatic properties, gender- and strain-specific expression, and role in renal 20-hydroxyeicosatetraenoic acid formation.. Biochem J.

[44] Kafoury RM, Pryor WA, Squadrito GL, Salgo MG, Zou X (1998). Lipid ozonation products activate phospholipases A2, C, and D.. Toxicol Appl Pharmacol.

